# A study on longitudinal relationship between ultrafine dust and the prevalence of depression

**DOI:** 10.1186/s12889-023-17375-z

**Published:** 2023-12-08

**Authors:** Kyu-Hyoung Jeong, Hayoung Park, Hyun-Jae Woo, Bo Kyung Kim, Ju Hyun Ryu, Seoyoon Lee

**Affiliations:** 1 Department of Social Welfare, Semyung University, 65 Semyung-ro, Jecheon, 27136 Republic of Korea; 2 Institute of Symbiotic Life-TECH, Yonsei University, 50 Yonsei-ro, Seodaemun-gu, Seoul 03722 Republic of Korea; 3 Seoul Metropolitan Council Health & Welfare Committee, 125 Sejong-daero, Taepyeongno 1(il)-ga, Jung-gu, Seoul 04519 Republic of Korea; 4 Graduate School of Social Welfare, Yonsei University, 50 Yonsei-ro, Seodaemun-gu, Seoul 03722 Republic of Korea; 5 Interdisciplinary Graduate Program in Social Welfare Policy, Yonsei University, 50 Yonsei-ro, Seodaemun-gu, Seoul 03722 Republic of Korea

**Keywords:** Ultrafine dust, Depression, Latent growth modeling, Longitudinal, Korea

## Abstract

**Background:**

Recently, the fine dust problem caused by rapid industrialization and science and technological development has emerged as a severe social issue worldwide. This also increases the interest in its effect on human life. In particular, there is a growing concern about the harm of fine dust in Korea.

**Methods:**

This study is based on the PM 2.5 data from 2017 to 2021 provided by Air Korea to estimate changes in ultrafine dust. In addition, the data from the Community Health Survey provided by the Korea Centers for Disease Control and Prevention (KCDC) from 2017 to 2021 were used to examine the effect between the change in ultra-fine dust and the prevalence of depression. A total of 229 local governments were included in the analysis. The Latent Growth Modeling was carried out to estimate the change in ultra-fine dust and the prevalence of depressions and verify the relationship between ultra-fine dust and the prevalence of depression.

**Results:**

The analysis result revealed that the ultra-fine dust concentration continued to decrease from 2017 to 2021. However, the depression prevalence increased from an average of 2.60% in 2017 to an average of 3.12% in 2021, suggesting the need for adequate and sufficient welfare policies for depression treatment. As a result of estimating the initial value and change rate of ultra-fine dust and depression prevalence, the higher the initial value of ultra-fine dust, the greater the decrease in ultra-fine dust. In terms of depression, the lower the initial value of the prevalence of depression, the larger the increase in depression prevalence.

**Conclusions:**

This study is significant in that it revealed the strong association of the longitudinal relationship between ultra-fine dust and depression, one of the biggest issues in Korea, by utilizing large-scale longitudinal data.

## Background

As fine dust has become a serious social issue in recent years as a result of rapid industrialization and the development of science and technology, interest in the impact of fine dust on human health has grown. According to the World Health Organization (WHO) International Agency for Research on Cancer, fine dust has been classified as a class 1 carcinogen since 2013. The fine dust not only affects respiratory and cardiovascular diseases but also cerebrovascular and nervous system diseases, resulting in worsened lung function and increased mortality rates [1, 2]. Further, long-term high level of fine dust has been reported to have adverse effects not only on physical health but also on mental health, such as depression and suicide risk [3–5]. Academic efforts are being made to empirically verify the relationship between fine dust and mental health in various contexts.

According to previous studies on the effects of fine dust on mental health, fine dust causes inflammation and oxidative stress as it enters the human body, which can affect the pathogenesis of depression [6]. In terms of fine dust, there are differences in the effect on the human body depending on its particle size [7]. The ultra-fine dust (Particle Matter; PM 2.5) with a diameter of 2.5*µ*m even affects the dopamine secretion of the nervous system in the brain, which may cause depression to be aggregated and suicide rates to increase [6, 8], In other words, considering that growth in depression symptoms due to fine dust can result in an increase in the suicide rate, which reflects the health of society, it is necessary to explore and intervene in the relationship between fine dust and depression at the local government level.

However, previous studies that explored the relationship between fine dust and depression have been restricted to the influence of fine dust on individual depression, which is limited in that they have not taken the regional prevalence of depression in a more macroscopic context into consideration. The relationship between fine dust and depression has been studied cross-sectionally and piecemealed, but no longitudinal study has explored this relationship. This study applied the methodology of Latent Growth Modeling to longitudinally estimate the changes in the prevalence of fine dust and depression at the regional level in Korea, allowing for an in-depth exploration of the rate of change between variables. The study aimed to verify the impact of the initial value and rate of change in the fine dust on the initial value and rate of change in the prevalence of depression. Through this, it is expected to expand the relationship between fine dust and depression as suggested in previous studies into a longitudinal and macroscopic context; it will enable the establishment of practical measures to reduce the prevalence of depression through intervention in the local government-level-centered on environmental factors. The research questions set to achieve the end are as follows.

1. What is the initial value and change rate of the prevalence of ultra-fine dust and depression?
2. What is the effect of the initial value and change rate of ultra-fine dust on the initial value and change rate of the prevalence of depression?

## Methods

### Data

This study is based on the PM 2.5 data from 2017 to 2021 provided by the Air Korea website (https://www.airkorea.or.kr/) to estimate changes in ultrafine dust. Air Korea, managed by the Korean Environment Corporation, is a homepage that publicly discloses real-time air quality information throughout the country, where people can get information on the fine dust. To confirm whether the change in ultra-fine dust affects the prevalence of depressions, data from the Community Health Survey provided by the Korea Centers for Disease Control and Prevention (KCDC) from 2017 to 2021 were used. This study analyzed 229 local governments that can estimate the prevalence of ultra-fine dust and depression from 2017 to 2021.

### Variables

#### Independent variable: ultra-find dust

The ultra-fine dust subject of this study is PM 2.5, which refers to fine dust with a diameter of less than 2.5*µ*m with a dust concentration smaller than 2.5/1000 mm. The annual average ultra-fine dust in each local government was used in this study.

#### Dependent variable: prevalence of depression

The depression of this study is diagnosed with the Korean version of the Patient Health Questionnaire-9 (PHQ-9), a self-report test that can screen for depression and its severity, which is a widely used tool in a clinical setting for its high validity and reliability [9]. PHQ-9 was developed by Kroenke, Spitzer and Williams [10] and comprised of 9 items asking about symptoms in the last two weeks based on nine diagnostic criteria for major depressive disorders. Respondents can answer with never (0 points), for several days (1 point), for more than a week (2 points), or for almost every day (3 points) for each item, and the total score ranges from 0 to 27 points. Previous researches suggested 10 points as a cut-off value for depression [10, 11]. This study also defined a PHQ-9 score of 10 or higher as having depression. The prevalence of depression was set as the percentage (%) of people with a total PHQ-9 score of 10 or higher for each local government.

### Statistical analysis

The analysis method and procedure used in this study to validate the longitudinal relationship between ultra-fine dust and the prevalence of depression are as follows. SPSS 27.0 and M-plus 8.0 programs were used for data handling and model analysis. First, descriptive statistical analysis was conducted to identify the characteristics of the main variables. Second,

Third, Latent Growth Modeling was carried out to estimate the change in ultra-fine dust and the prevalence of depressions and verify the relationship between ultra-fine dust and the prevalence of depression. Tucker-Lewis Index (TLI), Comparative Fit Index (CFI), and Root Mean Square Error of Approximation (RMSEA) were used to determine the fitness of the model. The research methodology of this study was reviewed by professors and scholars who hold doctoral degrees in nursing, public health, and related fields.

## Results

### Descriptive statistics

Ultra-fine dust continued to decrease from an average of 24.72*µ*g/m³(Standard Deviation; SD = 3.31) in 2017 to an average of 17.78*µ*g/m³(SD = 3.12) in 2021 (Table 1). The prevalence of depression increased from an average of 2.60% (SD = 1.31) in 2017 to an average of 3.12% (SD = 1.39) in 2021.

**Table 1:** Descriptive statistics

Years		Min	Max	Mean	SD
Ultra-fine dust	2017	15.17	35.72	24.72	3.31
	2018	14.91	31.79	22.87	3.18
	2019	12.63	32.63	22.59	4.01
	2020	10.28	25.21	18.56	3.02
	2021	10.93	25.17	17.78	3.12
Depression	2017	0.00	6.80	2.60	1.31
	2018	0.10	7.00	2.66	1.33
	2019	0.40	8.00	2.86	1.38
	2020	0.00	8.40	3.05	1.43
	2021	0.10	8.30	3.12	1.39

Note: Min = Minimum, Max = Maximum, SD = Standard Deviation

### Research model analysis

The potential growth model was analyzed into two stages. In the first stage, the Analysis of Unconditional Model was used to estimate the initial value and change rate of ultra-fine dust and depression prevalence. In the second stage, the relationship between ultra-fine dust and the prevalence of depressions was examined through the Analysis of Conditional Model based on the initial value and change rate obtained in the first stage.

#### Analysis of unconditional model

Before proceeding with the analysis of conditional model, an analysis of unconditional model was carried out to find out the changes in ultra-fine dust and the prevalence of depression (Table 2). To identify the optimal change pattern through the unconditional model, the no growth model and the Linear Growth Model were analyzed, respectively. The result shows that the goodness of fit of the Linear Growth Model better explains the relation between ultra-fine dust (χ^2^ = 57.071(*p* < .001), CFI = 0.905, TLI = 0.905, RMSEA = 0.089) and the prevalence of depression (χ^2^ = 31.651(*p* < .001), CFI = 0.937, TLI = 0.937, RMSEA = 0.077) than that of No Growth Model.

**Table 2:** Model fit of unconditional model

Model		χ^2^	*df*	CFI	TLI	RMSEA
Ultra-fine dust	No Growth Model	145.529^***^	13	0.738	0.753	0.143
	Linear Growth Model	57.071^***^	10	0.905	0.905	0.089
Prevalence in depression	No Growth Model	43.366^***^	13	0.884	0.892	0.101
	Linear Growth Model	31.651^***^	10	0.937	0.937	0.077

Note: ^***^p < .001, df = degree of freedom; CFI = Comparative Fit Index; TLI = Tucker-Lewis Index; RMSEA = Root Mean Square Error of Approximation

The analysis result of the finally selected unconditional linear growth model shows that the average of the initial ultra-fine dust values is 24.133(*p* < .001), and the variance is 8.886(*p* < .001), indicating the initial levels of ultra-fine dust differ among local governments (Table 3). The average change rate of ultra-fine dust is -1.593(*p* < .001), and the variance is 0.553(*p* < .001), showing that ultra-fine dust decreases over time, displaying the change rate of ultra-fine dust differs among local governments.

**Table 3:** Mean and variance of initial score and rate of change of unconditional model

Variables		Mean		Variance		Covariances
		Estimate	S.E.	Estimate	S.E.	
Ultra-fine dust	Initial Score	24.133^***^	0.244	8.886^***^	0.308	− 0.405^***^
	Rate of Change	-1.593^***^	0.062	0.553^***^	0.093	
Prevalence in depression	Initial Score	2.605^***^	0.085	1.046^***^	0.164	− 0.048^***^
	Rate of Change	0.115^***^	0.027	0.054^**^	0.017	

Note: ^**^p < .01, ^***^p < .001, S.E.=Standard Error

The average of the initial value of depression prevalence is 2.605(*p* < .001), and the variance is 1.046(*p* < .001), showing the initial value of depression prevalence varies among local governments. The average change rate of depression prevalence is 0.115(*p* < .001), and the variance is 0.054(*p* < .01), displaying that depression prevalence increases over time and showing that there is a difference in the change rate of depression prevalence among local governments.

The covariances of both the initial value and the change rate of ultra-fine dust and the prevalence of depressions were significant in a negative value. In other words, the local government with a higher initial ultra-fine dust value showed a more significant decrease in terms of ultra-fine dust than the local government with a lower initial ultra-fine dust value. In terms of depression prevalence, the local government with a higher initial prevalence value showed a smaller increase in depression prevalence than the local government with a lower initial prevalence value.

#### Analysis of unconditional model

In the analysis of conditional model, the effect of the initial value and change rate of ultra-fine dust on the initial value and change rate of depression prevalence was verified. The result of the analysis of conditional model goodness of fit was χ2 = 117.608(*p* < .001), CFI = 0.932, TLI = 0.919, RMSEA = 0.084, verified to have no problem in analyzing the model (Table 4).

**Table 4:** Path coefficient of study model

Path between Variables			Coef.	S.E.
Initial value of ultra-fine dust	→	Initial value of depression	0.118^***^	0.033
Initial value of ultra-fine dust	→	Change rate of depression	0.097^***^	0.027
Change rate of ultra-fine dust	→	Change rate of depression	0.029	0.015

Note: ^***^p < .001, Coef. = Coefficient; S.E.=Standard Error

**Fig. 1:**
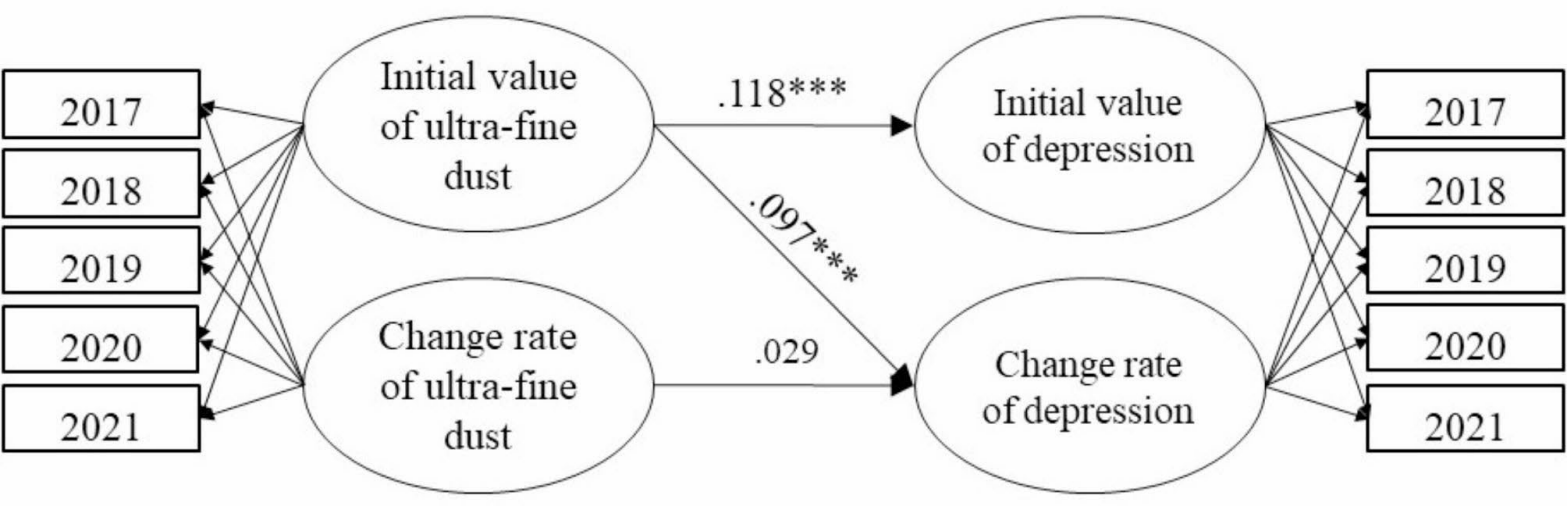
Result of the research model analysis

The initial value of ultra-fine dust has a significant effect on the initial value of the depression prevalence (Coef.=0.118, *p* < .001) and its change rate (Coef.=0.097, *p* < .001) (Fig. 1). In other words, the depression prevalence was high in the local governments with higher initial ultra-fine dust values and showed a drastic surge over time. However, the depression prevalence was low in the local governments with lower initial ultra-fine dust values and showed a gradual increase over time. On the other hand, the change rate of ultra-fine dust was found to have no significant effect on the change rate of the prevalence of depression.

## Discussion

The adverse effects of ultrafine dust on health have been a major concern in the research field. As air pollution worsens, there have been an increasing number of studies supporting the relationship between ultrafine dust and mental health. The growing body of research highlighted the impact of ultrafine dust on mental well-being [12, 13]. In particular, this study focuses on the relationship between ultrafine dust and depression. The association between ultrafine dust and depression is related to the biological mechanisms by which ultrafine dust particles affect the brain [14]. To be more exact, inhaled ultrafine dust can penetrate the nasal passages and enter the bloodstream, potentially affecting the nervous system. This direct influence on the neurological pathways related to stress and emotional regulation can have detrimental effects on human beings [15]. The relationship between ultrafine dust and depression has been examined and proved to correlate in the field of pathology. However, despite the increasing risk of continuous levels of ultrafine dust, the definitive relationship between ultrafine dust and depression is not yet clear. By pointing out the limitations of previous research, this study attempted to estimate the changes in ultra-fine dust and the prevalence of depression and verified the relationship between them.

To this end, air quality information from 2017 to 2021, reported by Air Korea, and managed by the Korea Environment Corporation, was utilized, and data on 229 local governments in Korea were collected. Latent Growth Modeling was carried out to estimate the change in ultra-fine dust and the prevalence of depressions and verify the relationship between ultra-fine dust and the prevalence of depression.

The analysis result revealed that the ultra-fine dust concentration continued to decrease from 2017 to 2021. Especially it decreased the most from an average of 22.59*µ*g/m³ in 2019 to an average of 18.56*µ*g/m³ in 2020, which may be the result of strong fine dust policies, such as the introduction of the first fine dust season management in December 2019 first time in Korea and strengthening the standards for air pollutant emissions at business sites [16]. Furthermore, the decrease in national energy consumption, ship arrival and departure, and air operations due to the COVID-19 situation may have positively affected the improvement of ultra-fine dust [17]. However, the depression prevalence increased from an average of 2.60% in 2017 to an average of 3.12% in 2021, suggesting the need for adequate and sufficient welfare policies for depression treatment. As a result of estimating the initial value and change rate of ultra-fine dust and depression prevalence, the higher the initial value of ultra-fine dust, the greater the decrease in ultra-fine dust. This may be due to discriminatory responses by each region, according to the survey on changes in ultra-fine dust concentration, as the ultra-find dust measurement equipment was installed in all cities (162 cities) in Korea from 2015 to 2020. In terms of depression, the lower the initial value of the prevalence of depression, the larger the increase of depression prevalence, showing that a preventive approach for the local governments with low depression prevalence may be required. The investigation also showed that the depression prevalence was high in the local governments with higher initial ultra-fine dust values and showed a rapid increase over time. This result supports some previous studies that the risk of depression may increase in people living in areas with high concentrations of ultra-fine dust [18–23].

This study concludes a longitudinal relationship between ultra-fine dust and depression. This suggests that ultra-fine dust reduction measures are required to reduce the depression prevalence in the region. However, as it is not easy to control and manage ultra-fine dust in reality, the region with high air pollution based on the current state of ultra-fine dust must be linked with practical measures to mitigate local depression prevalence. One example would be the ‘Urban Forest Creation Project’, carried out by the National Institute of Forest Science, which alleviated the risk of depression through various activities along with the ultra-fine dust reduction effects. Moreover, by utilizing the powerful environment monitoring system, local ultra-fine dust concentration must be monitored, and a priority and intensive depression prevention program for regions with high ultra-fine dust concentration must be developed. In addition, it is possible to consider a method that provides preemptive mental health services for regions where a potentially high prevalence of depression is predicted by considering trends in ultra-fine dust concentration by region over time. Furthermore, the management of the objective data on multifaceted policy efforts for ultra-fine dust-depression is needed.

This study is significant in that it revealed the strong association of the longitudinal relationship between ultra-fine dust and depression, one of the biggest issues in Korea, by utilizing large-scale longitudinal data. However, it has a limitation that this research could not control the external effect of COVID-19 on depression at the time. Also, the data on depression prevalence from the Community Health Survey this research is based on may have data bias issues caused by underreporting of depression. Specific individual features such as age or family relationships may have different influences on the longitudinal relationship between ultra-fine dust and depression. Further epidemiological studies are recommended to be carried out by supplementing these limitations. Furthermore, in this study, we examined the average annual concentration of ultrafine dust while considering the level of depression. However, to obtain more definitive results, it is recommended to devise alternative methods. In future studies, it is recommended to consider variables other than the average annual concentration of ultrafine dust. Additionally, although the analysis unit of this study was at the local government level, the dependent variable focused on the number of individuals with depression in each locality. While analyzing at the local government level is feasible, there were limitations due to utilizing measurements at the individual level. Therefore, it is recommended to devise methods that take depression into account at the local government level for following studies.

## Data Availability

The data sets analyzed in this paper are available publicly at the Air Korea website (https://www.airkorea.or.kr/).

## References

[1] Hetland R, Cassee F, Refsnes M, Schwarze P, Låg M, Boere A, Dybing E (2004). Release of inflammatory cytokines, cell toxicity and apoptosis in epithelial lung cells after exposure to ambient air particles of different size fractions. Toxicol in Vitro.

[2] Kumar D, Jugdutt BI (2003). Apoptosis and oxidants in the heart. J Lab Clin Med.

[3] Bakian AV, Huber RS, Coon H, Gray D, Wilson P, McMahon WM, Renshaw PF (2015). Acute air pollution exposure and risk of Suicide completion. Am J Epidemiol.

[4] Lim Y, Kim H, Kim J, Bae S, Park H, Hong Y (2012). Air pollution and depressive symptoms in elderly adults. Environ Health Perspect.

[5] Kim K-N, Lim Y-H, Bae HJ, Kim M, Jung K, Hong Y-C (2016). Long-term fine particulate matter exposure and major depressive disorder in a community-based urban cohort. Environ Health Perspect.

[6] Wang Y, Kloog I, Coull BA, Kosheleva A, Zanobetti A, Schwartz JD (2016). Estimating causal effects of long-term PM2. 5 exposure on mortality in New Jersey. Environ Health Perspect.

[7] World Health Organization (WHO). Ambient air pollution: A global assessment of exposure and burden of disease. 2016.

[8] Shin J, Park JY, Choi J (2018). Long-term exposure to ambient air pollutants and mental health status: a nationwide population-based cross-sectional study. PLoS ONE.

[9] Choi HS, Choi JH, Park KH, Joo KJ, Ga H, Ko HJ, Kim SR (2007). Standardization of the Korean Version of Patient Health Questionnaire-9 as a screening instrument for major depressive disorder. J Korean Acad Family Med.

[10] Kroenke K, Spitzer RL, Williams JB (2001). The PHQ-9: validity of a brief depression severity measure. J Gen Intern Med.

[11] Spitzer RL, Kroenke K, Williams JB, Group PHQPCS, Group PHQPCS (1999). Validation and utility of a self-report version of PRIME-MD: the PHQ primary care study. JAMA.

[12] Fan S-J, Heinrich J, Bloom MS, Zhao T-Y, Shi T-X, Feng W-R (2020). Ambient air pollution and depression: a systematic review with meta-analysis up to 2019. Sci Total Environ.

[13] Zeng Y, Lin R, Liu L, Liu Y, Li Y (2019). Ambient air pollution exposure and risk of depression: a systematic review and meta-analysis of observational studies. Psychiatry Res.

[14] Schraufnagel DE (2020). The health effects of ultrafine particles. Exp Mol Med.

[15] Zundel CG, Ryan P, Brokamp C, Heeter A, Huang Y, Strawn JR, Marusak HA. Air pollution, depressive and anxiety disorders, and brain effects: a systematic review. Neurotoxicology. 2022. 10.1016/j.neuro.2022.10.011 PMC10015654 36280190

[16] Fine dust seasonal. Management system ended, fine dust decreased [press release]. Sejong: Office for Government Policy Coordination Press Release; April 2020. p. 1.

[17] In 2020, the concentration of ultrafine dust was 19µg/m³, the lowest since the observation [press release]. Sejong: Ministry of Environment, 4 January 2021.

[18] Jo KH, Ryu SY, Han MA, Choi SW, Shin MH, Park J (2021). Cross-sectional associations between Particulate Matter (PM 2.5) and depression (PHQ-9) in the Elderly. J Health Inf Stat.

[19] Wang R, Liu Y, Xue D, Yao Y, Liu P, Helbich M (2019). Cross-sectional associations between long-term exposure to particulate matter and depression in China: the mediating effects of sunlight, physical activity, and neighborly reciprocity. J Affect Disord.

[20] Zhang Z, Zhao D, Hong YS, Chang Y, Ryu S, Kang D (2019). Long-term particulate matter exposure and onset of depression in middle-aged men and women. Environ Health Perspect.

[21] Borroni E, Pesatori AC, Bollati V, Buoli M, Carugno M (2022). Air pollution exposure and depression: a comprehensive updated systematic review and meta-analysis. Environ Pollut.

[22] Rajkumar RP (2023). The relationship between ambient fine particulate matter (PM2. 5) Pollution and Depression: an analysis of data from 185 countries. Atmosphere.

[23] Wu J, Grande G, Triolo F, Pyko A, Sjöberg L, Ljungman P (2023). Air pollution, social engagement, and depression in older adults: results from a Swedish population-based cohort study. Environ Pollut.

